# In Vitro Comparison of the Bioactivities of Japanese and Bohemian Knotweed Ethanol Extracts

**DOI:** 10.3390/foods9050544

**Published:** 2020-04-30

**Authors:** Lea Pogačnik, Tina Bergant, Mihaela Skrt, Nataša Poklar Ulrih, Jitka Viktorová, Tomáš Ruml

**Affiliations:** 1 Department of Food Science and Technology, Biotechnical Faculty, University of Ljubljana, Jamnikarjeva 101, 1000 Ljubljana, Slovenia; bergant.tina@gmail.com (T.B.); mihaela.skrt@bf.uni-lj.si (M.S.); natasa.poklar@bf.uni-lj.si (N.P.U.); 2 The Centre of Excellence for Integrated Approaches in Chemistry and Biology of Proteins, 1000 Ljubljana, Slovenia; 3 Department of Biochemistry and Microbiology, University of Chemistry and Technology Prague, Technicka 3, Prague 6, Czech Republic; jitka.prokesova@vscht.cz (J.V.); tomas.ruml@vscht.cz (T.R.)

**Keywords:** antioxidant activity, cellular antioxidant activity, polyphenols, anticancer activity, antimicrobial activity, antidiabetic activity, herbal medicine

## Abstract

Knotweed is a flowering plant that is native to temperate and subtropical regions in the northern hemisphere. We evaluated Japanese (*Reynoutria japonica* Houtt.) and Bohemian (*Fallopia x bohemica*) knotweed rhizome and flower ethanol extracts and compared them in terms of their biological activities. The specific polyphenols were identified and quantified using HPLC/DAD, and the antioxidant activity was determined using 2,2-diphenly-1-picrylhydrazyl (DPPH) and cellular antioxidant capacity assays. The anticancer activity was evaluated as the difference between the cytotoxicity to cancer cells compared with control cells. The antimicrobial activity was determined using bacteria and yeast. The antidiabetic activity was tested as the ability of the extracts to inhibit α-amylase. Both rhizome extracts were sources of polyphenols, particularly polydatin and (−)-epicatechin; however, the cellular assay showed the highest antioxidant capacity in the flower extract of *F. bohemica*. The PaTu cell line was the least sensitive toward all knotweed extracts. The flower extracts of both species were less toxic than the rhizomes. However, the activity of the tested extracts was not specific for cancer cells, indicating a rather toxic mode of action. Furthermore, all used extracts decreased the α-amylase activity, and the rhizome extracts were more effective than the flower extracts. None of the extracts inhibited bacterial growth; however, they inhibited yeast growth. The results confirmed that rhizomes of *Reynoutria japonica* Houtt. could become a new source of bioactive compounds, which could be used for the co-treatment of diabetes and as antifungal agents.

## 1. Introduction

*Reynoutria japonica* Houtt., known as Japanese knotweed, and hybrid *Fallopia x bohemica* (Chrtek & Chrtkova) J.P. Bailey, known as Bohemian knotweed, belong to the most aggressive groups of plant invaders [1,2]. *Reynoutria japonica* Houtt. and *F. x bohemica* are the most abundant knotweed species present in Slovenia [3] as well as in all of Europe [4].

All three knotweed species contain various bioactive substances, identified by several studies [5]. Among the three, *Reynoutria japonica* Houtt. has traditionally been used for the treatment of various inflammatory diseases in the countries of Eastern Asia [6]. It is well-known for its high content of resveratrol, a polyphenol with strong biological activity [7]. Its less known, but highly invasive relative, *F. x bohemica*, is known for its high growth rate. Both species provide various bioactive substances, such as stilbenes (e.g., resveratrol and polydatin) [8] and emodin [9], as well as different catechins [10] and carotenoids [11].

Studies suggested that *Reynoutria japonica* Houtt. contains bioactive substances, which can prevent tumor growth and metastasis to the lung [12], they can control melanoma cell proliferation [13] and can have a strong antimicrobial effect [14]. Antimicrobial activity was also confirmed for F. sachalinensis [15]. Researchers suggested that resveratrol supplementation may improve the antidiabetic effect against diabetes mellitus type 2 [16], which suggests possible antidiabetic properties of knotweed. Grzesik et al. [17] also found that two other polyphenols already determined in knotweed tissues, namely (+)-catechin and (−)-epicatechin, were the most effective compounds in protection against 2,2′-azobis(2-amidinopropane) dihydrochloride (AAPH)-induced erythrocyte hemolysis. The aim of our research was to compare the antioxidant, anticancer, antidiabetic, and antimicrobial activities of *Reynoutria japonica* Houtt. and *F. x bohemica* rhizome and flower ethanol extracts.

## 2. Materials and Methods

### 2.1. General Experimental Procedures

We used a UV-Vis spectrophotometer (Hewlett-Packard, model HP-8453, Palo Alto, CA, USA) to measure the absorbance. The fluorescence was determined using a SpectraMax MiniMax i3x Multi-Mode microplate reader (Molecular Devices, UK). HPLC system (Agilent Technologies, Inc., Wilmington, DE, USA), which consisted of a binary pump (Agilent 1260 Infinity model G1312B), the autosampler (model G1367E), and a diode array detector (model G4212B). The data signals were acquired and processed on a PC running the Agilent Chemstation (Agilent Technologies, Inc., Wilmington, DE, USA). HPLC analysis was carried out using a C18 column (Zorbax Eclipse Plus; 4.6 × 150 mm, 3.5 mm particle size; Agilent Technologies, Inc., Wilmington, DE, USA) and an analytical guard column (Agilent Eclipse XDB-C18; 4.6 × 12.5 mm, 5 mm particle size, Santa Clara, CA, USA).

2,2-diphenly-1-picrylhydrazyl (DPPH), Trolox (6-Hydroxy-2,5,7,8-tetramethylcroman-2-carboxy acid), chlorogenic acid (3-(3,4-Dihydroxyphenyl)prop-2-enoyl), malt extract broth, malt extract agar, *Luria Bertani* (LB) broth and *Luria Bertani* (LB) agar were brought from Sigma Aldrich, Germany. DCFH-DA (dichloro-dihydro-fluorescein diacetate), AAPH (2,2’-Azobis (2 amidinopropane) dihydrochloride), DMEM, (Dulbecco’s modified Eagle’s medium), EMEM, (Eagle’s minimum essential medium), trypsin solution, resazurin sodium salt, (7-hydroxy-3H-phe-noxazin-3-one10-oxide), α-amylase from porcine pancreas, (Type VI-B, ≥5 units/mg solid), and CNPG_3_, (≥96.0% (HPLC; 2-chloro-4-nitrophenly α—maltotrisoide)) were bought from Sigma Aldrich, USA. Trans-resveratrol (≥99% HPLC) was purchased from Fluka Analytical, Switzerland, Polydatin (≥95%); (+)-catechin hydrate (≥98%) and (−)-epicatechin (≥90%) (HPLC) were purchased from Sigma-Aldrich, Germany. The cell lines HeLa, PaTu, HEK 239T, and HepG2 were purchased from ATCC, USA.

### 2.2. Plant Material and Extraction Procedure

The rhizomes and flowers of *Reynoutria japonica* Houtt. and *F. x bohemica* were collected in September 2016 at two locations selected according to the records of previous samplings [18]. The samples were collected from around 9 p.m. to 11 a.m. in the morning in humid weather conditions at approximately 14 to 20 °C.

The fresh rhizomes were cleaned of mud and peeled. The flowers and rhizome peels were frozen with liquid nitrogen. The frozen plant material was put into a freeze drier (ALPHA 1-2/LD Plus, Christ, Germany) for 48 h to dehydrate. Lyophilized samples were finely ground in a coffee grinder. The freeze-dried samples were kept in closed test tubes in the freezer at −20 °C until used.

All lyophilized knotweed tissues were extracted twice with 96% ethanol [18,19] for 30 min on an ultrasound bath: rhizome’s peel (in the ratio 1:6) and flowers (in the ratio 1:12). Ethanol was evaporated using a rotary evaporator (R-210 Büchi, Switzerland). The obtained dry extract was dissolved in methanol to a mass concentration equal to 100 mg/mL for biological testing. These solutions were marked as stock extracts.

### 2.3. Identification and Quantification of Phenolic Compounds with HPLC/DAD

The HPLC separation of compounds in extracts was performed with a gradient elution: solvent A (1% HCOOH) and solvent B (100% methanol) with a flow rate 0.5 mL/min and a 10 μL injection volume [20]. The calibration curves were prepared for y in the concentration range from 0.5 to 15 µg/mL, 5 to 500 µg/mL, 5 to 500 µg/mL, and 25 to 200 µg/mL. The selected polyphenols were quantified by comparing the retention times of the relevant standards and comparing the spectrum of the chromatographic peaks at 280 nm (trans-resveratrol, polydatin) and 306 nm ((+)-catechin and (−)-epicatechin). The experiment was performed for two repetitions. The results were expressed in mg of individual polyphenol per gram of dry extract.

### 2.4. Antioxidant Capacity by DPPH

The assay is described in detail elsewhere [19]. Briefly, the reaction mixture was prepared as follows: fresh 0.11 mM DPPH (in methanol) was mixed with a 0 to 50 µL dose of 1.13 mM Trolox and 2% acetic acid to reach 50 µL. After 1 hour of incubation in the dark at room temperature, the absorbance was measured at 517 nm using a spectrophotometer. To determine the Trolox equivalent antioxidant capacity (TEAC), 5 µL of 10-times diluted stock extracts of each tissue were analyzed in the same way. The experiment was performed for two repetitions. The results were expressed in mmol of Trolox equivalents per gram of dry extract.

### 2.5. Cellular Antioxidant Activity Assay

The assay was realized in a 96-well plate according to [21]. Briefly, HepG2 cells were treated with 100 µL of 12.5 µg/mL DCFH-DA supplemented with the knotweed extracts (1 µg/mL). After 1 h of incubation at 37 °C, 5% CO_2_, the medium was removed and the cells were washed by PBS and finally, 100 µL of AAPH (0.164 mg/mL in PBS) was added to each well (except the blank wells). Immediately, the fluorescence (ex/em: 485/540 nm) was recorded in 5 min intervals for 1 h by the SpectraMax MiniMax i3x Multi-Mode microplate reader (Molecular Devices, UK). Luteolin was used as the negative control, whereas the cells in PBS (without AAPH addition) were tested as the positive controls. The values were obtained as an average of five repetitions and the results were expressed as the radical scavenging activity of knotweed extracts in percentage.

### 2.6. Cytotoxicity Assay

The cancer cell lines HeLa (human cervical adenocarcinoma derived cell line), HepG2 (human liver adenocarcinoma derived cell line), PaTu (human pancreatic adenocarcinoma derived cell line) and HEK 293T (human non-cancer epithelial kidney cell line) were cultivated and passaged using the standard procedures. All cell lines were obtained from the Collection of the Department of Biochemistry and Microbiology (DBM, UCT Prague, Czech Republic). Their sensitivity to commercial drugs was periodically controlled (the average IC_50_ of doxorubicin is about 40 µM). The cells of the 5th–30th passages were used for the experiments. The cells were seeded into the 96-well plates with appropriate medium in a concentration equal to 1 × 105 cells/mL. The cytotoxicity was realized as described previously [22]. Briefly, the cells were incubated with medium supplemented with the extracts (0.0625–1 mg/mL). After 48 h of incubation, the medium was removed, and the cells were washed with PBS. To determine the cell viability, 100 µL of resazurin solution (0.03 mg/mL) was added to each well. After 3 h incubation at 37 °C, 5% CO_2_, and 76% RH, the fluorescence was recorded at 560/590 nm (ex/em) by the microplate reader. The experiment was realized in two repetitions and the values are expressed as the average of the relative cell viability in percentage.

### 2.7. Antidiabetic Activity Assay

The extracts were diluted with methanol to concentrations from 0.001 mg/mL to 0.1 mg/mL. To 20 µL of the diluted extract, we added 60 µL of PBS and 10 µL of the α-amylase with a concentration of 0.8 mg/mL and incubated at room temperature (20 °C for 10 min) according to [23]. Afterwards, 10 µL of substrate CNPG3 (2-chloro-p-nitrophenyl-α-D-maltotrioside, 6.6 mg/mL) was added; after that, the absorbance (400 nm) was immediately recorded in 1 min intervals for 10 min. The positive control was prepared without the addition of the extract, the negative control contained no substrate. The values were obtained as an average of two repetitions and the results were expressed as an inhibition of α-amylase in percentage.

### 2.8. Antimicrobial Activity

The antimicrobial activity was tested against the spectrum of bacteria and fungi using the broth diffusion method. The following microorganisms were tested: bacteria *Escherichia coli*, *Pseudomonas aeruginosa*, *Proteus mirabilis*, *Proteus vulgaris*, *Enterococcus faecalis*, and *Bacillus megaterium*; and yeasts *Saccharomyces cerevisiae*, *Saccharomycodes ludwigii*, *Candida parapsilosis*, *Candida tropicalis*, and *Debaryomyces hanseii*. All strains were sensitive to commercial drug strains according to EUCAST (The European Committee on Antimicrobial Susceptibility Testing. Breakpoint tables for the interpretation of Minimal Inhibitory Concentrations (MIC) and zone diameters. EUCAST, Växjö, Sweden, Version 10.0, 2020). Both the antibacterial and anti-yeast activities were evaluated by the standard broth-dilution method using 96-well plates and Mueller Hinton (MH) broth or malt extract (ME) broth. The overnight microbial culture was diluted to a turbidity equal to 0.5 McFarland. After that, we performed the standard broth microdilution method recommended by EUCAST (The European Committee on Antimicrobial Susceptibility Testing). We used the breakpoint tables for the interpretation of MICs and zone diameters, (EUCAST, Växjö, Sweden, Version 10.0, 2020). We mixed and spread 10 µL of the diluted culture on the surface of the agar plate. After that, 10 µL of knotweed extract (100 mg/mL) was pipetted onto the surface of each agar plate. Methanol was used as the negative control. The plates were incubated at 37 °C (bacteria) and at 28 °C (yeasts) for 24 h. The antimicrobial activity was evaluated qualitatively by measurement of the inhibition zones (mm).

### 2.9. Data Procesing and Statistical Analysis

The data are presented as the averages of the appropriate number of repetitions, which are presented within the manuscript (n). The relative activity was evaluated as a percentage according to the formula: 100*(slope of sample fluorescence−average slope of PC)/(average slope of NC−average slope of PC). As the positive control (PC), the non-treated reaction was used. As the negative control (NC), the blank reaction was used. The IC_50_ values were determined using the software GraphPad Prism 7 and its function of nonlinear regression (Y = Bottom + (Top-Bottom)/(1 + 10^ ((LogIC-X)*HillSlope)). The statistical significance was checked with the Excel t-test function (two-tailed distribution, heteroscedastic type). One-way analysis of variance (ANOVA) was used followed by Duncan’s post hoc test (*p* < 0.05) to show the differences between the groups. For ANOVA, the software Statistica version 12 was used (Tibco Software Inc., Tulse, OK, USA).

## 3. Results and Discussion

### 3.1. Identification and Quantification of Phenolic Compounds

**Figure 1:**
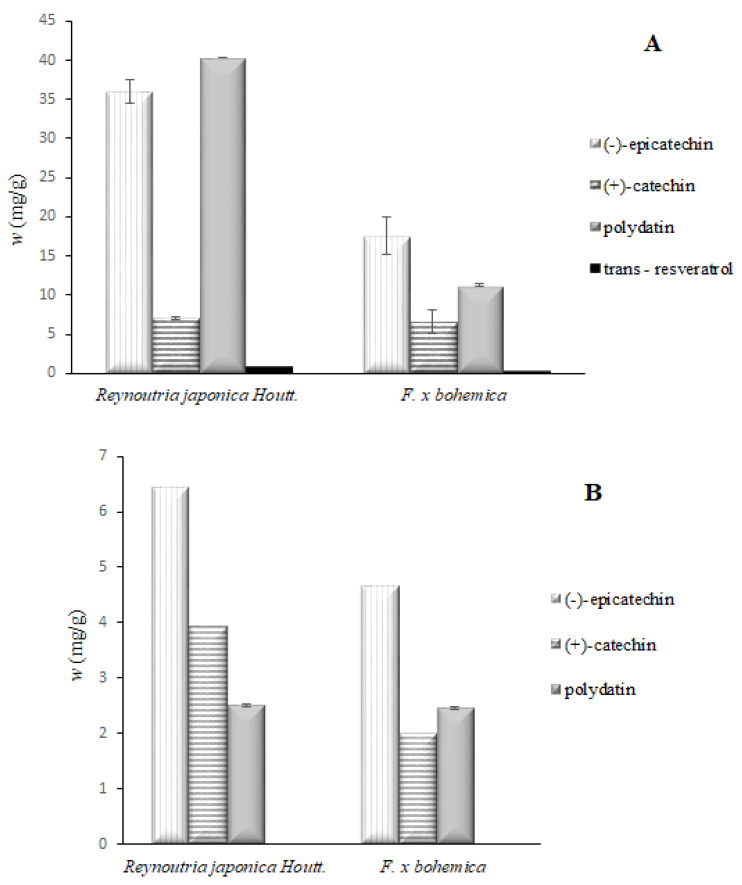
Mass fractions (*w*) of the investigated compounds in rhizome (**A**) and flower (**B**) extracts expressed in a mass of substance (in mg) in a gram of dry extract.

Based on the calibration curves of selected compounds (trans-resveratrol, polydatin, (−)-epicatechin, (+)-catechin), the contents of individual compounds were determined in all the extracts used in this study. Among stilbenes, trans-resveratrol has been the most widely studied for its effect on human health [24], and catechins were determined in spring sprouts of *Fallopia* species [25]. The highest contents of all investigated phenolic compounds were found in rhizomes (Figure 1A), whereas in the flowers (Figure 1B), the contents were considerably lower or not detectable at all. In the samples of *Reynoutria japonica* Houtt, higher levels of polydatin (40.3 mg/g) and (−)-epicatechin (26.1 mg/g) were determined, compared to trans-resveratrol (0.87 mg/g) and (+)-catechin (7.06 mg/g). All the investigated compounds were also determined in *F. x bohemica*, but in smaller quantities, whereas the contents of polydatin, (+)-epicatechin, trans-resveratrol, and (+)-catechin were 11.3 mg/g, 17.5 mg/g, 0.46 mg/g, and 6.56 mg/g, respectively.

**Figure 2:**
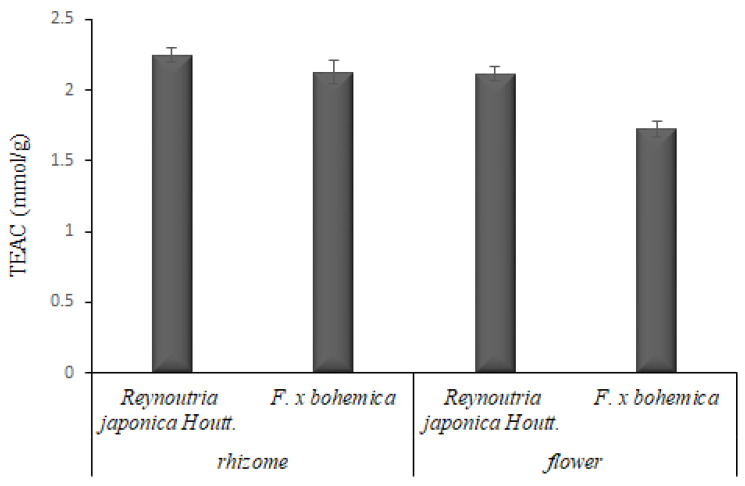
Antioxidant activity of knotweed extracts expressed as the Trolox equivalents (Trolox equivalent antioxidant capacity (TEAC), mmol/g). The data are presented as the average of two repetitions with the standard deviation.

**Figure 3:**
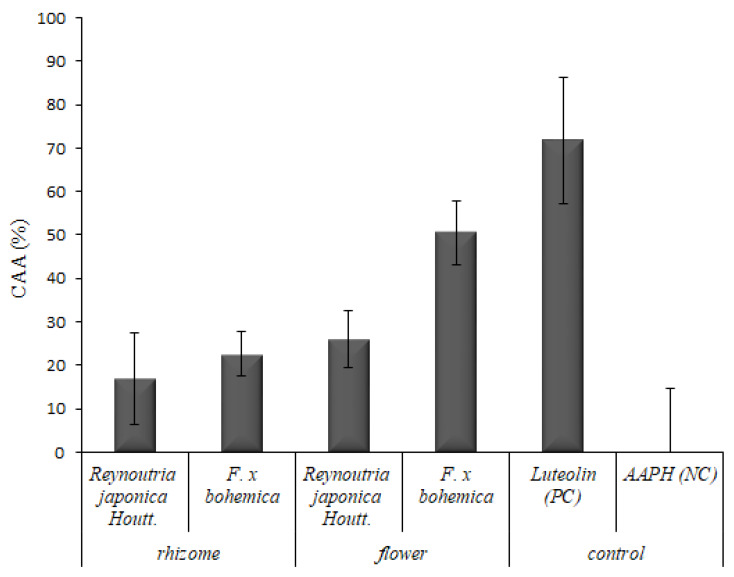
Cellular antioxidant activity (CAA) of knotweed extracts (γ = 0.001 mg/mL). Luteolin was used as a positive control (PC) and 2,2′-azobis(2-amidinopropane) dihydrochloride (AAPH) as a negative control (NC). Data are presented as the average of five repetitions with the standard deviation.

In the extracts obtained from *Reynoutria japonica* Houtt, flowers only (−)-epicatechin (6.45 mg/g), (+)-catechin (3.93 mg/g), and polydatin (2.5 mg/g) were determined. The quantities of (−)-epicatechin, (+)-catechin, and polydatin were lower in *F. x bohemica* at 4.67 mg/g, 1.99 mg/g, and 2.45 mg/g, respectively. Trans-resveratrol was not detected in any of the flower extracts. The results showed that the distribution of different polyphenols greatly differed across different tissues. The concentrations of (+)-catechin were similar in both tested tissues, whereas the quantities of other determined compounds were much lower in flowers compared to in rhizomes. This fact leads to the conclusion that, in the flowers, other antioxidants (not determined in our study) contribute to the relatively high antioxidant capacity determined in the further experiments (Figure 2 and Figure 3). In the study of [10], quercetin was detected in the flowers in addition to (+)-catechin and (−)-epicatechin. The flowers contained higher amounts of total quercetin than the stems.

A similar study showed that the concentration of polydatin in *Reynoutria japonica* Houtt. rhizome extracts was 10.33 mg/g, which is four times less than our values [8]. However, they found a concentration of 3.47 mg/g for trans-resveratrol, which is about four times more than our values. The results of both studies are difficult to compare, as different extraction solvents were used and the results were expressed in two different ways, per gram of dried ethanol extract (our study) and per gram of whole lyophilized knotweed tissue [8]. The ratio between trans-resveratrol and its glycosylated form, polydatin, varies not only by the location but also likely by the time period of the sample collection.

Another study [26] showed that the average concentration of polydatin was seven times higher in *Reynoutria japonica* Houtt. extracts than the concentration of trans-resveratrol, whereas our rhizomes were almost 50 times richer in polydatin compared to trans-resveratrol. However, the polydatin is more resistant to enzymatic oxidation than trans-resveratrol, which is water-soluble and penetrates the cell passively [27]. We can confirm that, by our extraction procedure, considerable amounts of the glycosylated form of resveratrol (polydatin) were preserved and low concentrations of trans-resveratrol can possibly be attributed to degradation during the handling of samples and extraction procedure.

Another interesting 3-year field study was performed by [28], who observed that the concentrations of different antioxidants, namely trans-resveratrol, resveratroloside, polydatin, and emodin decreased during the growth period in the aboveground biomass; and simultaneously, the concentrations in the belowground biomass increased, when the plant started to accumulate important storage substances (e.g., starches, sugars, and proteins as well as bioactive substances) for winter rest. This can explain the higher levels of antioxidants (up to six-times higher) determined in the roots compared to the flowers, as the plant material in our study was collected in September.

### 3.2. Antioxidant Capacity

In this study, two different methods for evaluation of antioxidant capacity of knotweed extracts, namely DPPH scavenging capacity assay and cellular antioxidant activity (CAA) assay, were used.

#### 3.2.1. DPPH Scavenging Capacity Assay

In the rhizome extracts, a higher antioxidant capacity was determined compared to the flower extracts with the *p* value equal to 0.015 for *Reynoutria japonica* Houtt. and 0.210 for *F. x bohemica* (Figure 2). The TEAC of rhizome extracts from both knotweed species were similar, namely 2.25 mmol/g for *Reynoutria japonica* Houtt. and 2.13 mmol/g for *F. x bohemica*. The flower extracts had lower TEAC values, namely *Reynoutria japonica* Houtt. 2.12 mmol/g and *F. x bohemica* 1.73 mmol/g.

In a previous study [19], the overall antioxidant capacity (determined by four different analytical methods) of different tissues of three knotweed species (*Reynoutria japonica* Houtt., *F. x bohemica* and *F. sachalinesis*) was the highest for the flowers of *Reynoutria japonica* Houtt. (0.71 mmol TEAC/g) and the flowers of *F. x bohemica* (0.40 mmol TEAC/g) compared to the rhizomes of *Reynoutria japonica* Houtt. (0.55 mmol TEAC/g) and the rhizomes of *F. x bohemica* (0.25 mmol TEAC/g), which is the opposite to our results. However, in our study, the values of antioxidant capacity are up to six-times higher. This observation could be attributed to the fact that in our study TEAC is expressed per gram of dry extract and not to the initial lyophilized powder that also contains the ethanol insoluble matter as in the cited work. Other important differences include the extraction solvent (96% ethanol used in this study and 50% ethanol in study of [19]) as well as the fact that in our study only the peel of rhizomes was used, whereas the other study used the whole rhizome, which is a much less rich source of antioxidants.

#### 3.2.2. Cellular Antioxidant Activity (CAA) Assay

Previously, research demonstrated that several compounds, which are able to scavenge the radicals in the classical biochemical assay, do not act as antioxidants in living cells. There may be several reasons for this. The compounds can be insoluble in the cultivation medium; they can poorly penetrate across the membrane or can be metabolized to non-active metabolites. Therefore, the cellular antioxidant activity is biologically more relevant as it includes several aspects of living organisms, such as the bioavailability and first-pass metabolism. To evaluate the antioxidant capacity in the cells, all diluted rhizome and flower extracts (1 µg/mL), obtained from *Reynoutria japonica* Houtt. and *F. x bohemica* were used to determine the CAA in human hepatocellular adenocarcinoma derived cells (HepG2). As it can be seen in Figure 3, the flower extract of *F. x bohemica* shows the highest antioxidant capacity among the tested extracts (*Reynoutria japonica* Houtt. rhyzome—*p* = 0.007, *Reynoutria japonica* Houtt. flowers—*p* = 0.066, *F. bohemica* rhizome—*p* = 0.037). The rhizome extracts of *Reynoutria japonica* Houtt. and *F. x bohemica* possessed the lowest antioxidant activity of all the tested extracts (*p* = 0.647).

The results of CAA do not correlate with the previously presented results obtained by the DPPH assay. The CAA method gave the highest values for rhizome extracts and the lowest values for flower extracts, which is opposite to the results of the CAA assay. The reason for this can be attributed to the fact that the DPPH assay belongs to the antioxidant capacity methods that are based on the transfer of hydrogen between the free radicals and the antioxidant. This method is a valid indicator of the antioxidant activity of a dietary substance, as the antioxidants directly react with radicals [29,30], but cannot provide the evidence of in vivo antioxidant activity when consumed. As explained above, the CAA assay developed by [31] is more biologically relevant than the classical biochemical assays [29]. The ability of chemical antioxidant activity assays to predict in vivo activity is often questioned for a number of reasons. Some are performed at non-physiological pH and temperature and none take into account the bioavailability, uptake, and metabolism of the tested compounds [31].

### 3.3. Cytotoxicity Activity

In order to investigate the possible toxic effect of knotweed extracts on the cells, the ability of extracts to lyse the cytoplasmic membranes of red blood cells was checked with a hemolytic test. None of the knotweed extracts showed toxic activity against the red blood sheep cells (results not shown) up to the concentration of 100 mg/mL. Further, we determined whether different concentrations of rhizome and flower extracts of *Reynoutria japonica* Houtt. and *F. x bohemica* affected the viability of cancer cells (HeLa, HepG2, and PaTu) and the non-cancer cell line (HEK 293T).

The results demonstrated (Table 1) that the PaTu cell line was the least sensitive to selected knotweed extracts among the tested cancer cell lines. All the tested extracts at the concentration of 0.5 mg/mL and higher (except flower extract from *Reynoutria japonica* Houtt. towards the PaTu cell line) showed 100% inhibition of both the cancer and non-cancer cell lines used in this study. The HeLa line was the most sensitive among the tested cancer cell lines and as sensitive as the control cell line. The rhizome extracts of *Reynoutria japonica* Houtt. and *F. x bohemica* were more toxic compared to the flower extracts. However, none of the tested extracts showed anticancer activity. Rather than specific inhibition of cancer cells, the extracts showed basic toxicity.

To the best of our knowledge, no data regarding the potential anticancer activity of knotweed extracts has been published so far. However, the review by [32] noted that trans-resveratrol, which is also present in the rhizomes of knotweeds, as shown in our study, possesses a large spectrum of pharmacological properties. The research demonstrated that trans-resveratrol affects all stages of carcinogenesis (initiation, promotion, and progression) by modulating the signal transduction pathways that control cell division. Thus, we can speculate that trans-resveratrol or its derivative polydatin, present in rhizome extracts of both knotweed species tested in this study, might have potential effects on the growth inhibition of cancer cell lines. However, other cell lines and other extraction procedures should be tested in order to confirm these speculations. Further studies would have to be performed in order to reduce the toxicity of higher concentrations of knotweed extracts towards non-cancer cell lines as well.

### 3.4. Antidiabetic Activity

In order to determine the antidiabetic activity of selected extracts, the α-amylase activity was measured as a decrease of 2-chloro-p-nitrophenyl-α-D-maltotrioside (CNPG3) substrate after the addition of different concentrations of extracts (0.001–0.1 mg/mL) obtained from flowers and rhizomes of *Reynoutria japonica* Houtt. and *F. x bohemica*.

The obtained α–amylase inhibition curves (Table 2) show the dose-dependent manner: the higher the applied concentration of all tested extracts, the higher the inhibition of α–amylase. However, considerable differences were observed among extracts from different species and tissues. Rhizome extracts from both tested species decreased the α–amylase activity in whole concentration range more efficiently than the flower extracts. At the highest extract concentration (0.1 mg/mL), the enzyme was completely inhibited by *Reynoutria japonica* Houtt. rhizome and *Reynoutria japonica* Houtt. flower extracts, whereas the other two extracts inhibited the enzyme only slightly less. Both extracts of *Reynoutria japonica* Houtt. were more active in α-amylase inhibition in comparison to both extracts of *F. x bohemica.* Thus, the rhizome extracts from *Reynoutria japonica* Houtt. were the best inhibitors of α–amylase within the whole tested concentration range and it can therefore be considered as a good source of substances with antidiabetic properties.

The inhibition of α-amylase and also α-glucosidase using polyphenol-rich functional foods is a novel policy to regulate the carbohydrate metabolism and supplementary and nutraceutical treatment for diabetes mellitus. Recent studies have shown that both (+)-catechin and (−)-epicatechin can effectively inhibit α-amylase (Rasouli et al. 2017, Kong et al. 2018). As shown in Figure 1, the rhizomes of both knotweed species are up to 7 times richer in (−)-epicatechin and (+)-catechin and 16 times richer in polydatin compared to flowers, which can be the reason for their better performance in antidiabetic assays. Moreover, *Reynoutria japonica* Houtt. extracts contain a higher amount of both biologically active compounds in comparison to *F. x bohemica*.

### 3.5. Antimicrobial Activity

The results of our study showed that none of the selected extracts up to the concentration of 100 mg/mL possessed any antimicrobial activity against the selected bacteria (*E. coli*, *P. aeruginosa*, *P. mirabilis*, *P. vulgaris*, *E. faecalis*, or *Bacillus megatherium*). The extracts showed activity against yeasts (*S. cerevisiae*, *S. ludwigii*, *C. parapsilosis*, *C. tropicalis*, and *D. hanseii*). The rhizome extracts showed stronger inhibition potential towards the majority of the yeast cultures compared to the flower extracts, which coincides with our results of the antioxidant capacity in vitro and antidiabetic activity. The rhizome extracts possessed strong inhibition towards *S. ludwigii* and *C. parapsilosis*, whereas the flower extracts demonstrated only a mild effect. The least sensitive yeast toward all knotweed extracts was *D. hanseii*.

In a similar study [14], where five common foodborne bacteria (*Bacillus cereus*, *Listeria monocytogenes*, *Staphylococcus aureus*, *E. coli*, and *Salmonella anatum*) were treated with crude extract of *Reynoutria japonica* Houtt. rhizomes, they showed potent antibacterial properties. However, their extraction procedures were different. More recently, Zhang et al. [33], similarly to our results, showed that the *Reynoutria japonica* Houtt. extract was able to inhibit the growth of the yeast *Candida albicans*. This yeast occurs in almost 17% of patients treated in intensive care units. They also found antimicrobial activity toward both bacterial strains (*A. baumannii* and *S. aureus*) and fungal strains (*A. fumigatus*).

## 4. Conclusions

We confirmed that the rhizomes and flowers of *Reynoutria japonica* Houtt. and *F. x bohemica* can be considered as novel sources of bioactive substances that can help in the prevention of diabetes and the spreading of fungal infections. The rhizome extracts more efficiently decreased the activity of α-amylase, compared with the flower extracts. We also demonstrated that knotweed extracts can be used as anti-yeast agents as they inhibited the growth of several food contaminating and pathogenic yeast strains. Based on the significant anti-yeast activity, we suggest the potential application of knotweed extracts in dermocosmetics as the antifungal components of creams. Similarly, for their antidiabetic activity, the extracts could find applications in insulin supplement therapy, inhibiting the polysaccharide cleavage to form glucose and maltose. Based on our analysis, further research could find other possible bioactive substances in addition to the ones identified in our study and study their bioactive effects, using both in vitro as well as in vivo animal models.

## Figures and Tables

**Table 1:** The concentrations [µg/mL] that halved the viability (IC_50_) of cancer (PaTu, HeLa, HepG2) and control (HEK 293T cell line) cells.

	Rhizome Extract		Flower Extract	
	*Reynoutria Japonica* Houtt.	*F. x Bohemica*	*Reynoutria Japonica* Houtt.	*F. x Bohemica*
HEK 293T	36.7 ± 4.9 ^ab^	24.9 ± 7.6 ^a^	92.4 ± 8.5 ^c^	54.7 ± 8.2 ^b^
PaTu	182.2 ± 48.5 ^a^	228.0 ± 37.3 ^a^	430.6 ± 135.8 ^b^	311.7 ± 58.9 ^ab^
HeLa	36.9 ± 4.2 ^a^	45.8 ± 7.0 ^a^	90.2 ± 13.9 ^b^	59.0 ± 6.1 ^a^
HepG2	63.0 ± 9.0 ^a^	63.7 ± 7.6 ^a^	277.3 ± 41.9 ^c^	174.7 ± 26.9 ^b^

The data are presented as the average of four repetitions ± standard error of the mean. Letters indicate the differences between the groups (one-way analysis of variance (ANOVA) followed by Duncan’s post hoc test, *p* < 0.05) within one cell line. The different cell lines were evaluated independently on each other. Statistically significant levels are denoted with different letters.

**Table 2:** The concentrations [µg/mL] that halved the α-amylase activity (IC_50_).

Plant Tissue	Species	IC_50_ [µg/mL]
Rhizome	*Reynoutria japonica* Houtt.	0.17 ± 0.02 ^a^
	*F.* x *bohemica*	6.5 ± 2.5 ^b^
Flower	*Reynoutria japonica* Houtt.	8.2 ± 0.4 ^bc^
	*F.* x *bohemica*	25.1 ± 13.3 ^c^

The data are presented as the average of two repetitions ± the standard error of the mean. Letters indicate the differences between the groups (ANOVA followed by Duncan’s post hoc test, *p* < 0.05). Statistically significant levels are denoted with different letters.
